# Asymmetrical Dimer Photonic Crystals Enabling Outstanding Optical Sensing Performance

**DOI:** 10.3390/nano13030375

**Published:** 2023-01-17

**Authors:** Hicham Mangach, Youssef El Badri, Abdelhamid Hmima, Abdenbi Bouzid, Younes Achaoui, Shuwen Zeng

**Affiliations:** 1 Light, Nanomaterials Nanotechnologies (L2n), CNRS-ERL 7004, Université de Technologie de Troyes, 10000 Troyes, France; 2 Laboratory of Optics, Information Processing, Mechanics, Energetics and Electronics, Department of Physics, Moulay Ismail University, B.P. 11201, Zitoune, Meknes 50000, Morocco

**Keywords:** asymmetric dimer PhCs, Fano resonance, high-quality factor, hybrid DNA

## Abstract

The exploration of the propensity of engineered materials to bring forward innovations predicated on their periodic nanostructured tailoring rather than the features of their individual compounds is a continuous pursuit that has propelled optical sensors to the forefront of ultra-sensitive bio-identification. Herein, a numerical analysis based on the Finite Element Method (FEM) was used to investigate and optimize the optical properties of a unidirectional asymmetric dimer photonic crystal (PhC). The proposed device has many advantages from a nanofabrication standpoint compared to conventional PhCs sensors, where integrating defects within the periodic array is imperative. The eigenvalue and transmission analysis performed indicate the presence of a protected, confined mode within the structure, resulting in a Fano-like response in the prohibited states. The optical sensor demonstrated a promising prospect for monitoring the DNA hybridization process, with a quality factor (QF) of roughly $1.53\times 10^{5}$ and a detection limit (DL) of $4.4\times 10^{-5}$ RIU. Moreover, this approach is easily scalable in size while keeping the same attributes, which may potentially enable gaze monitoring.

## 1. Introduction

Engineered materials have significantly advanced the science of wave manipulation over the past decade [1]. Indeed, the notion delineated in the seminal work of Eli Yablonovitch, who initially designed synthetic materials to control the spontaneous emissions of light through periodically changing the dielectric permittivity [2], has been expanded upon to bring forward a versatile assortment of devices: waveguides, energy localization in a flat state, and biosensor agents, among others [3,4,5]. Recently, the monumental challenge of the COVID-19 pandemic has shed light on the critical necessity of biosensors in the constant battle against that outbreak. Within this scope, a continuous pursuit to improve both the accuracy and efficiency of diagnosis in the current health care system is underway, and the biosensors community is harnessing advancements made in nanotechnology to bring forward breakthroughs in lab-on-chip, label-free, and real-time response diagnostic technologies [6,7,8,9]. While monitoring chemical interactions is the main goal, striking a balance between cost, overall performance, and convenience of use remains highly desirable. It is worth emphasizing that strong coupling between the optical waves and the high-affinity biomolecules is consequential to enable enhanced detection of the analyst [10]. Historically, the leaky mode of a planar waveguide was the cornerstone of many early biosensing technologies, which were applied to a wide range of applications, including disease detection and protein identification [11]. Barshilia et al. experimentally demonstrated that a planar waveguide-based sensor may reach a resolution of up to $5.65\times 10^{-4}$ [12]. Moreover, triggering the whispering gallery mode, whereby a reciprocation of energy occurs between a microring or microdisk cavity and a linear waveguide, has been thoroughly investigated due to the high-quality factors supported by these systems, which is a straightforward outcome of the extended interaction lifespan with the guided photons within the resonator [5,13,14]. The resonance type of Wood’s anomaly, commonly known as guided mode resonance, which Lord Rayleigh described as a tangential scattering component lying on the grating’s surface at the resonance [15], was also proposed as a prospect sensing technology by Wang et al. [16]. Zhou et al. revealed through numerical investigation a figure of merit (FOM) of 5709 with grating waveguides based on guided mode resonance, which results from a supplying empirical relationship between the grating period and the depth of the groove to induce a narrow linewidth in the transmission [17]. On the other hand, silicon photonic crystal (PhC) sensors are a promising platform due to their miniaturized nature, high sensitivity (S), precise detection limit (DL), as well as their viable operation at visible wavelengths [18,19]. A DL of 0.002 RIU is achieved with the PhC cavity, whereas the nano-ring’s DL is one order of magnitude better [20,21]. In regard to heterostructure cavity-based PhCs, Di Falco et al. achieved a substantially low DL of approximately $7.8\times 10^{-6}$ RIU and a high quality factor (QF) of about $5\times 10^{4}$ [22]. Furthermore, considering the versatility of integrating these conventional PhCs within the context of the other methods stated above, new avenues for building advanced photonic devices emerge. For instance, the incorporation of porous silicon into PhCs was shown to result in a fourfold enhancement of the DL for proteins, polymer layers, and bulk liquids [23,24]. In other respects, bi-dimensional nanowire PhCs based on semiconductor materials have demonstrated the ability to not only increase charge mobility but also to act as a viable alternative for trapping and tuning Fabry–Perrot oscillations [25,26]. Additionally, due to their high aspect ratio, these nanowires PhC provide considerable surface-to-volume interaction space, which is particularly promising for biosensor applications [27]. Chen et al. demonstrated a highly sensitive plasmonic sensor based on dual-side polished PhC fibers with a refractive index (RI) resolution of approximately $9.39\times 10^{-6}$ [28]. However, this approach is known to be highly dissipative, since it promotes the scattering process at the metal’s surface, which results in increased ohmic losses. Thus, it generates additional heat that might significantly reduce the sensor’s lifetime. Recently, it has been demonstrated that the exceptional points found at the phase singularities that correspond to the bifurcation properties of non-Hermitian degeneracy may be used to improve the sensitivity of resonant optical structures to external perturbations [29]. Currently, the resonance phenomenon is widely believed to be the foundation for developing the next generation of optical sensors. Particularly, the high-quality factors exhibited during the Fano resonance have been the driving force behind several breakthroughs in the discipline of optics, including the interpretation of the multipolar scattering characteristics of spherical particles, the enhancement of surface plasmon resonance through metallic arrays, the production of plasmon-induced transparency phenomena, and the breaking of the symmetry of split-ring metamaterials [30,31,32]. The process of this resonance was described by Ugo Fano in 1961 as the outcome of the constructive and destructive interferences between localized discrete states and continuum states, which leads to an asymmetrical response that sets it apart from the standard Lorentzian shape [33,34]. Fano resonance sensors are remarkable for their ability to measure minute changes in RI, making them ideal transducers for phase or intensity interrogation that transform the electrical signal produced by the interaction of biorecognition elements and analytes [35]. In this letter, we report on an optical sensor with an asymmetric dimer structure (a dimer unit cell with asymmetrically spaced inclusions) that exhibits a high QF. Most notably, as opposed to the cavity-based PhC, the sensing is accomplished without resorting to the creation of any defect within the structure, thereby facilitating the fabrication process. This can open up brand-new paths in the realm of photonic biosensor devices. The structure is constructed of silicon due to its high RI and low energy consumption at biological windows, making it the material of choice for the target application. Silicon microelectronic chip manufacture has strongly shaped the development of several sectors, including telecommunications, light emitters, detectors, and effective in vitro diagnosis tools. This is due to its compatibility with low energy applications, abundance, and convenience of etching, especially compared to other semiconductor materials [36]. Toward that end, we employ a numerical model based on the Finite Element Method (FEM) to delineate both the energy momentum diagram and the transmitted signature of the transverse electric (TE) configuration. A computation of the phase interrogation is also provided to explore the selectivity and detection limit of our proposed design.

## 2. Materials and Methods

**Figure 1:**
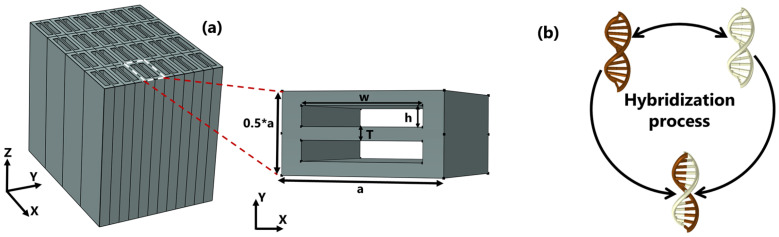
(**a**) Schematic design of a three-dimensional array and an in-plane overview of the dimer PhC with a period of a=1 μm; w=750 nm and h=125 nm are the width and height, respectively; *T* is gap spacing ranging from 100 to 30 nm. (**b**) An illustration of the hybridization process of two single DNAs.

Figure 1a depicts the schematic design of the biosensor as well as the in-plane aspect of the unit cell. The unit cell has lateral dimensions of *a* and 0.5a along the *x* and *y* directions, respectively, inside which two air gaps are aligned to form an asymmetrical pattern. The structure is made of silicon, and the gaps are filled with the test samples, with RIs of $n_{Si}$ and $n_{sample}$, respectively. Throughout the simulation we carried out, the dielectric properties of gaps were accounted for as the refractive indices of the bio-molecular target under consideration, which is hybridized DNA in this case. The single DNAs are initially diluted in water; therefore, the RI of the full piece does not differ much from that of water. Furthermore, the dispersion properties of silicon are incorporated into the numerical model to provide a detailed description of the device.

The suggested structure’s dispersion and transmission responses are evaluated using eigenvalue and harmonic computations based on the FEM using the proprietary software COMSOL Multiphysics (COMSOL, Inc., Burlington, MA, USA) version 6.0. Furthermore, since the Fano-like response is generated along the *x*-axis, we examine unidirectional propagation with the wavenumber *k* swept along ΓX. We consider an incoming plane wave with *z*-polarization at normal incidence that propagates along the *x*-axis, as indicated in Equation (1). All the dimer-based PhCs studied have symmetry translations in the (X, Y) plane while being homogeneous in the polarization direction. The fundamental aspect of periodicity within the unit cell invokes the Bloch–Floquet theorem, which states that the solution is a plane wave modulated by a periodic function with the same period as the crystal periodicity [37].

(1) $$\vec{E_{z}}\left(x\right)=E_{z_{0}}e^{-j(wt-k_{0}x)}$$

(2) $${\left[\begin{array}{ccc} \varepsilon_{xx} & 0 & 0\\ 0 & \varepsilon_{yy} & 0\\ 0 & 0 & \varepsilon_{zz} \end{array}\right]}^{-1}\left\{\begin{array}{c} 0\\ 0\\ \frac{\partial^{2}E_{z}}{\partial x^{2}} \end{array}\right\}-k_{0}^{2}\left\{\begin{array}{c} 0\\ 0\\ E_{z} \end{array}\right\}=0$$

(3) $$\varepsilon^{-1}\left(\vec{r}\right)=\sum_{\vec{G}}C_{m}e^{i\vec{G}\vec{R}}$$

where $\varepsilon_{xx}=\varepsilon_{yy}=\varepsilon \left(r+a\right)$, $\varepsilon_{zz}=\varepsilon \left(r\right)$ are the in-plane dielectric permittivity, which follows the same periodicity as the unit cell and the out-plane permittivity. $k_{0}=2\pi /\lambda_{0}$ and $E_{z_{0}}$ are the wavenumber in vacuum and the magnitude of the incident electric field. The set of reciprocal lattice vectors and the Fourier components of the periodic dielectric function are expressed by $\vec{G}$ and $C_{m}$, respectively.

**Figure 2:**
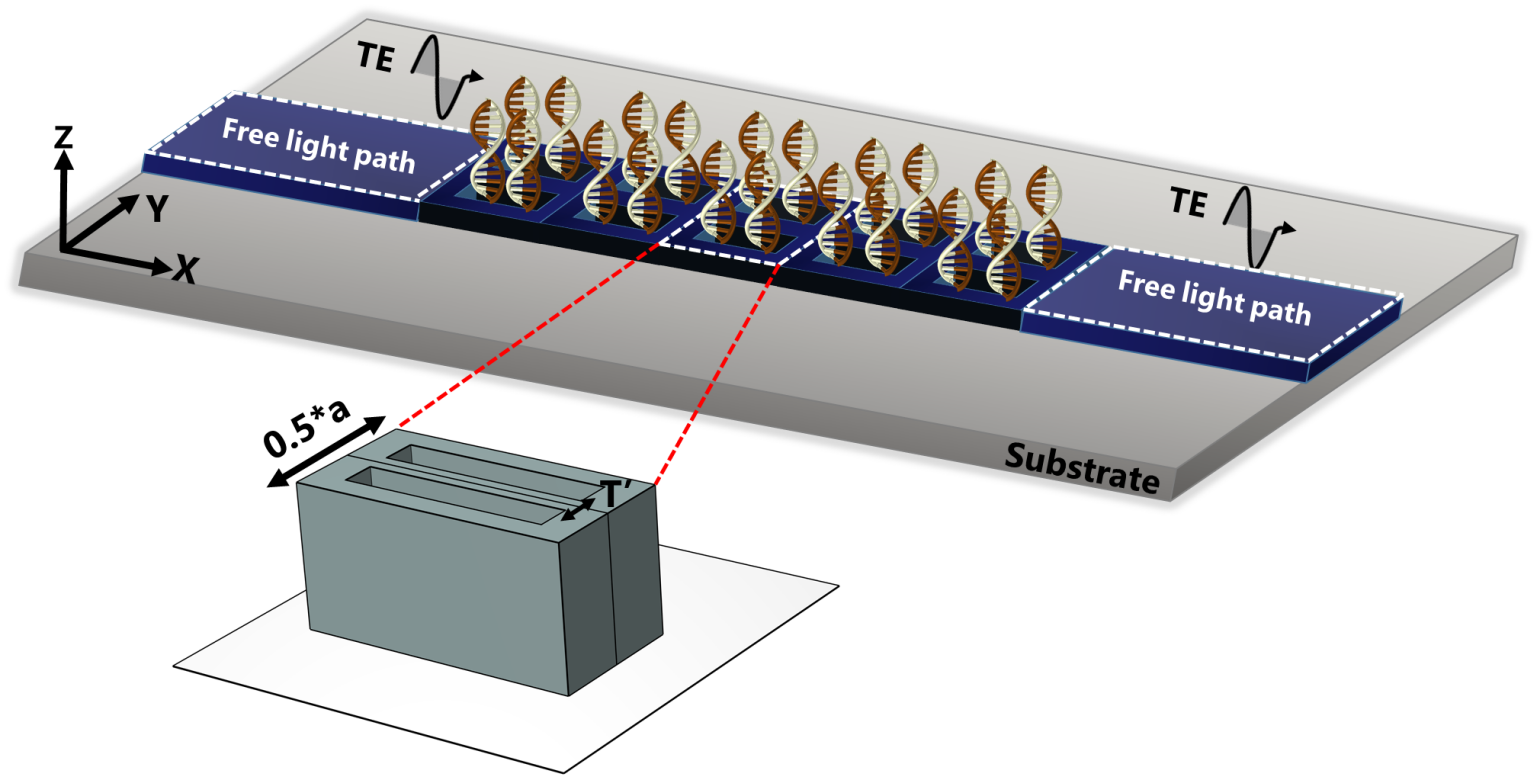
Illustration of the transmission measurement procedure that entailed irradiating the dimer PhC with a TE-polarized wave traveling from the left along the ΓX direction of the irreducible Brillouin zone, the energy is recorded at the right side for different stages of DNA hybridization.

For the sake of outlining a general understanding of the behavior of electromagnetic waves within the structure, the photonic dispersion diagram is obtained by solving the eigenvalue problem delineated in Equation (2). The transmission signatures are acquired by driving *z*-polarized plane waves from the left side of the structure while measuring the energy transmitted on the right side, as illustrated in Figure 2.

In order to quantify the performance of our optical sensor, multiple factors must be examined. The most significant ones are the sensitivity (S), QF, FOM, and DL. The sensitivity is defined as the ratio between the smallest resolved wavelength shift and the change in RI that caused it, as described by Equation (4). This parameter describes the intensity of the interaction between the target solution and the fraction of the electromagnetic waves immobilized within the structure. The FOM measures the precision accuracy, which is inversely proportional to the full width at half maximum (FWHM), as shown in Equation (5). Furthermore, the amount of energy stored in any resonator is commonly recognized to be determined by the amount of energy localized inside it, which is properly represented by the quality factor, as displayed in Equation (6) [38]. It reflects the degree of coupling between the external stimulus and the active target inside the biological window; the higher this parameter, the more favorable the interaction conditions. As shown in Equation (7), the detection limit takes into account all the aspects influencing detection performance, which is inherently correlated to the spectral resolution and sensitivity of the system [20,39].

(4) $$S=\frac{\Delta \lambda_{r}}{\Delta n_{target}}\left(\mathrm{nm}\;/\;\mathrm{RIU}\right)$$

(5) $$\mathrm{FOM}=\frac{S}{\mathrm{FWHM}}\left({\mathrm{RIU}}^{-1}\right)$$

(6) $$\mathrm{QF}=\frac{\lambda_{r}}{\mathrm{FWHM}}$$

(7) $$\mathrm{DL}=\frac{\lambda_{r}}{\left(10Q\cdot S\right)}\left({\mathrm{RIU}}^{-1}\right)$$

where $\lambda_{r}$ is the wavelength of resonance. $\Delta \lambda_{r}$ and $\Delta n_{target}$ are variations in the resonance wavelength and the RI, respectively.

## 3. Results and Discussion

**Figure 3:**
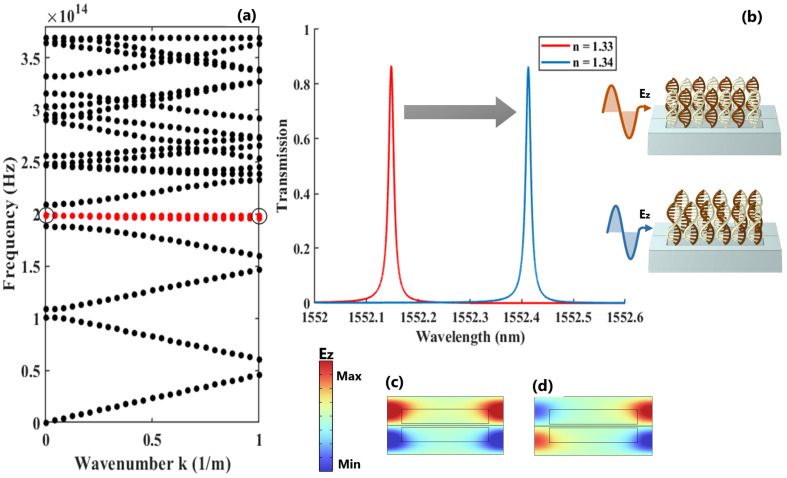
(**a**) The energy-momentum diagram of dimer PhC along the ΓX direction, indicating the allowed TE modes throughout this direction; (**b**) the spectral shift of the resonance when the refractive index of the sample changes from 1.33 to 1.34; (**c**,**d**) the representation of the electric field distribution at the resonance and anti-resonance points, respectively.

The eigenvalue analysis of the out-of-plane electric field for the proposed asymmetric dimer PhC exhibits a flat mode with near-zero energy transport, as highlighted by the red line in Figure 3a. The associated transmission response of this confined mode during the initial and final stages of the hybridization process is depicted in Figure 3b. Additionally, the electric field distributions at resonance and anti-resonance denoted by the circles in the dispersion diagram are shown in Figure 3c and Figure 3d, respectively. It is noteworthy that this asymmetric signature in the transmission response is a direct outcome of the asymmetry incorporated within the unit cell.

**Figure 4:**
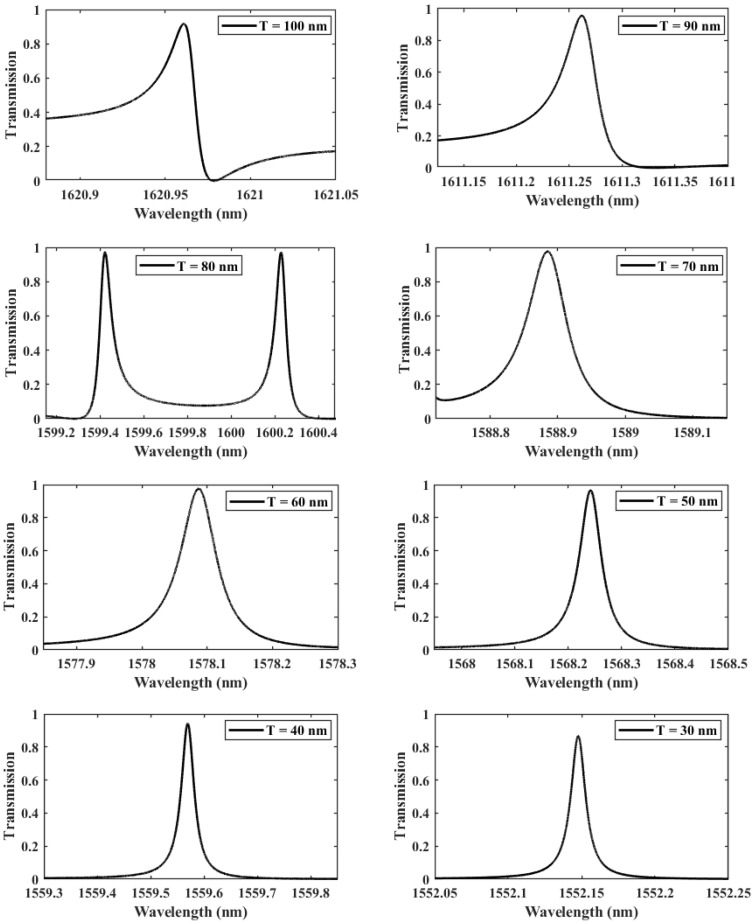
The evolution of Fano resonance transmission patterns as the gap separation *T* is spanning from 100 to 30 nm.

In what follows, an in-depth study to optimize the design of the structure was carried out, in which we led a sequence of transmission computations with different values of the thickness separating the two gaps *T*, from 30 to 100 nm, as seen in Figure 4. The adequate thickness value is determined accordingly based on the evolution of the quality factor, which measures the coupling between the localized mode and the hybridized DNA. The curve of the transmission response becomes sharper for small thicknesses (low values of *T*), with a significant increase in the QF from 23,719 at T=100 nm to $1.33\times 10^{5}$ at T=30 nm, reflecting that the amount of energy stored inside the asymmetric dimer unit cell is enhanced. Consequently, the asymmetry introduced is beneficial to the interaction environment and considerably increases the biosensor’s effectiveness, as shown in Table 1. Nevertheless, it is evident that the rise of the QF is not linear, as it drops significantly between the gap thicknesses of 80 nm and 60 nm. This can be explained by the weak coupling regime between the localized mode and the propagating mode within the crystal. As a result, three separate coupling regimes may be distinguished: the first between 100 and 80 nm, the second between 80 and 60 nm, and the third between 60 and 30 nm. The first two regimes correspond to the typical coupling between the flat mode and the propagation modes present inside the crystal, in which the mismatch between the two modes increases, resulting in energy localization losses. In contrast, the last regime, in which the localized mode occurs alone in the forbidden state, results in a massive amount of energy being stored in the structure with no interference with the other modes.

**Figure 5:**
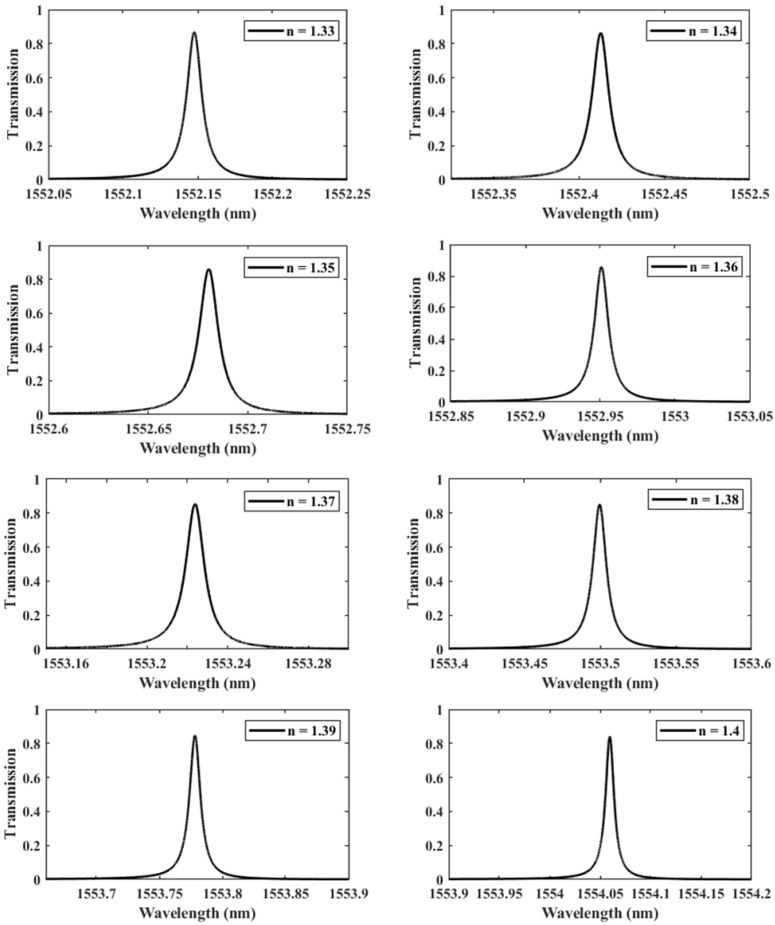
The shifting of the resonance wavelength with respect to the refractive index *n* of the sample varied from 1.33 to 1.4, demonstrating different phases of DNA hybridization.

**Figure 6:**
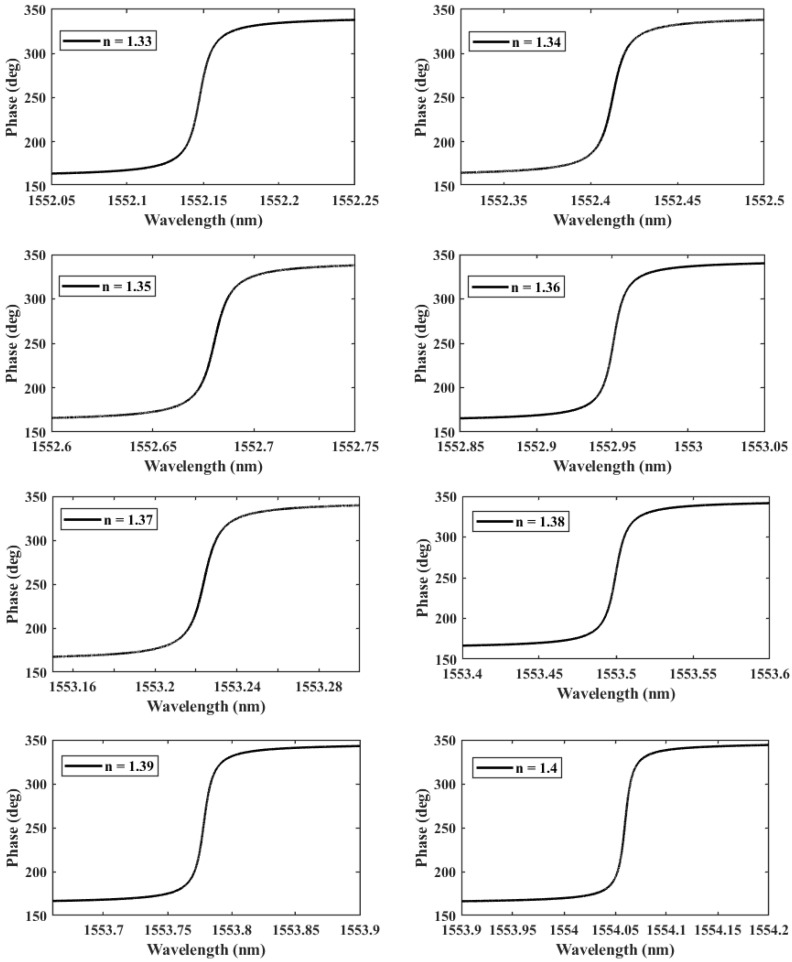
The phase of the transmitted wave as a function of the RI *n* during the formation of a double-DNA.

**Figure 7:**
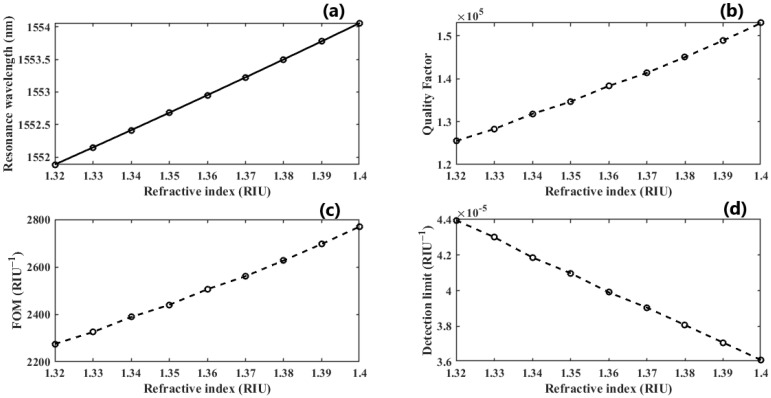
(**a**) The linear operative regime of dimer PhCs illustrating sensitivity; (**b**,**c**) the quality factor and FOM growth as the RI of the sample increases; (**d**) the DL as the double-DNA refractive index changes.

We further investigate the case of T=30 nm since it provides the highest QF. We perform another parametric analysis on the refractive indices corresponding to single and double DNA in order to monitor the various stages of the DNA hybridization process. We irradiate the proposed structure with *z*-polarized plane waves at normal incidence, as indicated in Figure 2. Accordingly, a series of transmitted responses are recorded at the output while the refractive index is swept from 1.32 to 1.4, as delineated in Figure 5, which highlights the Fano resonance’s red-shift. Figure 6 depicts the phase analysis of the transmission spectrum that indicates a $180^{\circ }$ phase change, which is a salient feature of the Fano resonance and is also known as the pi-resonance: the phase abruptly switches between two out-of-phase points (resonance and anti-resonance). Figure 7a demonstrates the shift in resonance wavelength as the refractive index changes, i.e., the sensitivity. Notably, Figure 7b shows that the quality factor progressively increases as the refractive index of the sample increases. In other words, the higher the RI, the better the phase matching between localized waves and bioanalyte. Similarly, the FOM, which is defined as the ratio of sensitivity to the FWHM, is another physical indication used to measure the sensor’s capacity to detect minor fluctuations at resonance. Figure 7c indicates that the FOM increases with RI, reaching a maximum of 2770 ${\mathrm{RIU}}^{-1}$. Finally, Equation (7) was used to calculate the device’s ability to detect minute, resolvable RI changes, $\mathrm{DL}=4.4\times 10^{-4}$. It is worth mentioning that these results are well within the linear operation regime, which is often viewed as an essential characteristic for any sensor. Moreover, these findings show that our device is far from the saturation regime, enabling more flexibility in detecting any fluctuations beyond the regime of the optical properties explored within the scope of this investigation.

Finally, we contextualize our findings with the relevant literature. Table 2 summarizes recent research conducted in the field of biophotonic sensors, which include planar stacked layers (1D PhCs), topology-induced high-sensitivity photonic sensors, and 2D-photonic biodevices based on morphological cavities and cavity-coupled waveguides. Our approach entailed providing a new alternative based on an asymmetric dimer unit cell with a protected, confined mode within the structure. This is not accessible in conventional photonic crystals, where a punctual or linear defect is required to realize such selectivity. Asymmetric dimer PhCs demonstrated outstanding performance, rivaling the current state of the art in optical biosensor devices. We emphasize that this work is merely a proof of concept, and further numerical and experimental work is still needed.

## 4. Conclusions

Engineered materials have brought extraordinary innovations to different disciplines of science. To that end, we employed numerical analysis to study the optical properties of a dimer PhC design, wherein symmetry alterations were introduced. This structure’s optical properties have been examined using eigenvalue and transmission measurements, and the possibility of using it to monitor the hybridization process of DNA has been explored. A QF of $1.53\times 10^{5}$ was obtained. The suggested structure provides a novel alternative that supports the Fano resonance without resorting to the creation of any defects, which is unavoidable in conventional PhCs. Furthermore, these findings are modular and can be extended to explore other subjects, including gas identification.

## Figures and Tables

**Table 1:** The progression of the spectral resonant frequency, quality factor, and transmittance properties of dimer PhC as a function of gap thickness *T*.

Thickness (nm)	Resonance Wavelength (nm)	Transmission (%)	Quality Factor
100	1621	91.97	23,719
90	1611.3	95.54	37,194
80	1599.4, 1600.23	97.34	23,492, 25,975
70	1588.9	97.61	23,600
60	1578.1	97.46	5833.4
50	1568.2	96.62	31,405
40	1559.6	94.3	53,593
30	1552.1	86.7	133,403

**Table 2:** Comparative analysis of cavities, topology, and heterogeneous structure in recent 1D and 2D PhCs sensors.

Sensor Configuration	Quality Factor (%)	FOM	Detection Limit	Ref
1D APC	$1.51\times 10^{5}$	$2.6\times 10^{4}$	$10^{-6}$ $\;{\mathrm{RIU}}^{-1}$	[40]
1D topology	$10^{5}$	$4.967\times 10^{4}$	−	[41]
2D linear cavity	$2.5\times 10^{4}$	3700	$1.25\times 10^{-5}$ $\;{\mathrm{RIU}}^{-1}$	[42]
–	$2.676\times 10^{4}$	–	110 pg/mm^2^	[43]
2D nanoring cavity	$3\times 10^{3}$	–	0.2 fg	[21]
2D hyterostructure cavity	$5\times 10^{4}$	–	$7.8\times 10^{-6}$ $\;{\mathrm{RIU}}^{-1}$	[22]
2D rings-slot cavity	$1\times 10^{7}$	–	$8.75\times 10^{-5}$ $\;{\mathrm{RIU}}^{-1}$	[44]
Our work	$1.55\times 10^{5}$	2770	$4.4\times 10^{-5}$ $\;{\mathrm{RIU}}^{-1}$	

## Data Availability

The data presented in this study are available upon request from the corresponding author (rshuwen.zeng@cnrs.fr).
